# Deep learning-based effusion-synovitis volume measured on MRI is associated with osteoarthritis progression: A longitudinal analysis of data from the osteoarthritis initiative

**DOI:** 10.1016/j.ocarto.2026.100847

**Published:** 2026-06-27

**Authors:** Adrian A. Marth, Felix Liu, Ethan Pan, Sevtap T. Ulas, John A. Lynch, Alexandra S. Gersing, Nancy E. Lane, Michael C. Nevitt, Charles E. McCulloch, Thomas M. Link, Gabby B. Joseph

**Affiliations:** a Department of Radiology and Biomedical Imaging, University of California, San Francisco, USA; b Department of Radiology, Balgrist University Hospital, Zurich, Switzerland; c Faculty of Medicine, University of Zurich, Zurich, Switzerland; d Department of Epidemiology and Biostatistics, University of California, San Francisco, USA; e Department of Internal Medicine, U.C. Davis Health, Sacramento, CA, USA

**Keywords:** Osteoarthritis, Knee, Synovitis, Biomarkers, Magnetic resonance imaging, Deep learning

## Abstract

**Objective:**

To investigate whether the 48-month change in effusion-synovitis volume (ΔESV) is associated with concurrent knee osteoarthritis progression, and to compare these associations with those of semiquantitative change in effusion-synovitis using the MRI Osteoarthritis Knee Score (ΔMOAKS).

**Design:**

In this study using data from the Osteoarthritis Initiative conducted from 02/2004-10/2015, deep learning-based measurements of knee ESV were derived from baseline and 48-month follow-up knee MRI (n = 2469). OA outcomes included change of Kellgren-Lawrence (KL) grade, Whole-Organ Magnetic Resonance Imaging Score (WORMS) and its subscales (meniscus, bone marrow edema-like lesions [BMELL], cartilage), and symptom progression by the Western Ontario and McMaster Universities Osteoarthritis Index (WOMAC). Associations of ESV change (ΔESV) with OA outcome changes were evaluated using spline-based regression models. Effect sizes were reported as interdecile differences (IDDs; 90th vs 10th percentile of the exposure). To compare the associations between ΔESV and ΔMOAKS, differences in IDD (ΔIDD) were estimated.

**Results:**

ΔESV was significantly associated with ΔKL grade, ΔWORMS_Total_, ΔWORMS_Meniscus_, ΔWORMS_BMELL_, ΔWORMS_Cartilage_, and ΔWOMAC (all p < 0.001). Compared with ΔMOAKS, ΔESV demonstrated significantly stronger associations for ΔKL grade (ΔIDD = 0.22; p = 0.002), ΔWORMS_Total_ (ΔIDD = 0.21; p = 0.008), and ΔWORMS_Meniscus_ (ΔIDD = 0.20; p = 0.026), whereas differences were non-significant for ΔWORMS_BMELL_ (p = 0.51), ΔWORMS_Cartilage_ (p = 0.25), or ΔWOMAC (p = 0.19).

**Conclusions:**

Longitudinal change in ESV was associated with concurrent imaging-based and symptomatic osteoarthritis progression over 48 months. Compared with changes in MOAKS scores, ΔESV showed stronger associations with imaging-based OA outcomes. These findings underscore the potential of MRI-based ΔESV as an osteoarthritis imaging biomarker, while further studies are needed to establish its predictive and clinical utility.

## 1 Introduction

In knee osteoarthritis (OA) research, there is a strong interest to identify and validate modifiable biomarkers that can facilitate early disease detection and/or predict disease progression [[1], [2], [3], [4], [5]]. Among the various risk factors implicated in OA onset and progression, synovitis is increasingly recognized as a driver of structural disease progression [[6], [7], [8], [9]] and associated with increased pain [10,11].

Joint effusion and synovial thickening are characteristic imaging features of knee synovitis. However, intra-articular fluid and synovial tissue cannot be reliably differentiated on non-contrast-enhanced MRI, and therefore, these findings are collectively referred to as “effusion-synovitis” [12]. Effusion-synovitis is routinely evaluated using well-established semiquantitative scoring systems, such as the MRI Osteoarthritis Knee Score (MOAKS) [13], which have been previously used to investigate the association between synovitis and OA progression [[6], [7], [8], [9]]. However, these ordinal scoring systems have inherent limitations, including limited reproducibility and sensitivity to change [[14], [15], [16]]. Recently, a deep learning-based method for automated quantification of knee effusion-synovitis volume (ESV) on MR images has been developed and validated [17]. However, the association of quantitative ESV with OA progression, as well as its sensitivity to change, remains unclear.

Therefore, the objectives of this study were to investigate whether the 48-month change in deep learning-based effusion-synovitis volume (ΔESV) is associated with imaging and clinical outcomes of concurrent OA progression, and to compare the strength of associations with OA progression between ΔESV and semiquantitative change in effusion-synovitis on the MOAKS (ΔMOAKS).

## 2 Design

### 2.1 Participant selection

Data for this study were obtained from the Osteoarthritis Initiative (OAI) database (https://nda.nih.gov/oai). The OAI is a large, multicenter, prospective, Health Insurance Portability and Accountability Act (HIPAA)-compliant cohort study established to identify and validate imaging and clinical biomarkers of knee OA. All study protocols andprotocol amendments were approved by the institutional review boards of each participating center, and written informed consent was obtained from all participants.

**Fig. 1:**
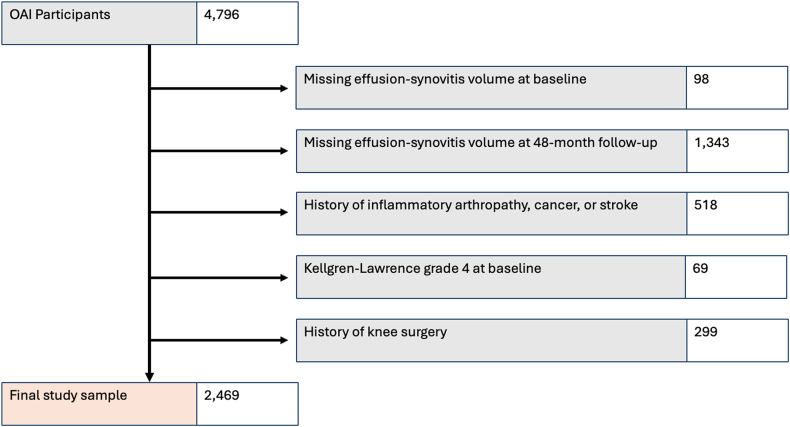
Participant selection process. *OAI, Osteoarthritis Initiative*.

From the 4796 individuals enrolled in the OAI, the following exclusion criteria were applied to the present study: (i) Missing right-knee ESV measurements at baseline or 48-month follow-up, (ii) history of self-reported inflammatory arthritis, cancer, or stroke at baseline or follow-up, (iii) evidence of end-stage radiographic OA of the right knee (Kellgren-Lawrence [KL] grade 4) at baseline and (iv) history of knee surgery at baseline. A history of cancer was used as an exclusion because of potential disease or treatment-related systemic inflammatory effects, and because cancer-related morbidity and mortality could contribute to differential loss to follow-up. Stroke was used as an exclusion because resulting neurological impairment can alter gait, limb loading, and physical activity, which might influence joint structural progression independently of synovitis. Using these criteria, a sample of 2469 participants was included in the final analysis (Fig. 1).

### 2.2 MRI protocol

Baseline and 48-month follow-up MR images of the right knee were acquired at participating sites using cross-calibrated 3.0-T MR systems (Magnetom Trio, Siemens Healthineers, Erlangen, Germany). The sequences analyzed in the present study included sagittal three-dimensional dual-echo steady-state (DESS) sequences with water excitation (repetition time 4.7 ms, echo time 16.3 ms, field of view 14.0 cm, in-plane resolution 0.37 × 0.46 mm, slice thickness 0.7 mm, flip angle 25°, bandwidth 185 Hz/pixel) as well as sagittal intermediate-weighted fat-suppressed fast spin-echo sequences (repetition time 3200 ms, echo time 30 ms, field of view 16.0 cm, in-plane resolution 0.36 × 0.51 mm, slice thickness 3.0 mm, flip angle 180°, bandwidth 248 Hz/pixel). The detailed imaging acquisition parameters that were previously applied in the OAI have been described in previous publications [18].

### 2.3 Deep learning-based effusion-synovitis volume assessment

ESV was quantified at baseline and follow-up in milliliters (mL) using a previously developed and validated fully automated deep learning-based pipeline [19] built upon neural network foundation models [20,21]. The model was trained on manually annotated sagittal DESS MRI from 101 knees, with annotations performed by two readers under expert musculoskeletal radiologist supervision (TML, with more than 20 years of experience) using dedicated annotation software. Cases were selected using stratified sampling to ensure balanced representation across effusion-synovitis severity levels. The pipeline integrates object detection and instance segmentation by combining a You Only Look Once (YOLOv11, ultralytics.com)-based detector with a Segment Anything (SAM, ai.meta.com)-based segmentation model. Manual segmentation masks were converted into bounding boxes to supervise YOLOv11 training and to serve as prompts for SAM-based segmentation.

Model inputs consisted of three-channel images incorporating (i) 2D DESS slices, (ii) spatially registered intermediate-weighted fat-suppressed fast spin-echo images, and (iii) mean-intensity projection DESS images. Training data included all slices containing effusion-synovitis annotations, supplemented with a random sample of 25% of slices without annotations to improve robustness. To enhance spatial generalizability, images and corresponding masks were reformatted into axial and coronal orientations in addition to the native sagittal plane, and models were trained independently in each plane, yielding a total of six trained models.

The dataset was randomly partitioned into training, validation, and test sets using an 80/10/10 split, with the test set held out exclusively for performance evaluation. The fully automated inference pipeline first performed multi-planar object detection, followed by plane-specific instance segmentation. Segmentations generated in axial and coronal planes were subsequently transformed into the sagittal plane and consolidated to produce final volumetric masks. To reduce misclassification of adjacent structures with similar signal characteristics, automatically generated cartilage, meniscus, and bone masks were subtracted from the predicted effusion regions. Further details and segmentation performance of the deep learning-based pipeline are shown in Supplementary Table S1. Two representative cases with good and suboptimal deep learning-based segmentations compared to manual reference segmentations are shown in Supplementary Figure S1.

### 2.4 Semiquantitative assessment of effusion-synovitis

Semiquantitative assessment of effusion-synovitis at baseline and follow-up was performed using the effusion-synovitis subscale of MOAKS, a well-established classification system widely used in OA research [13]. Semiquantitative gradings (n = 1682) were available from a previous study [22] and performed by two board-certified radiologists (RM, CN) under the supervision of a musculoskeletal radiologist with more than 20 years of experience (TML). Effusion-synovitis was graded on axial reformations of DESS images as follows: grade 0 = physiologic amount of fluid; grade 1 = small - fluid continuous in the retropatellar space; grade 2 = medium - with slight convexity of the suprapatellar bursa; grade 3 = large - evidence of capsular distention [13].

### 2.5 Outcome measures - radiographic assessment of osteoarthritis

Radiographic OA at baseline and follow-up was assessed using the KL grading system. The KL scale ranges from grade 0 to grade 4 and is defined as follows: grade 0 = no radiographic features of osteoarthritis; grade 1 = doubtful joint space narrowing and possible osteophytic lipping; grade 2 = definite osteophyte formation with possible joint space narrowing; grade 3 = multiple osteophytes, definite joint space narrowing, sclerosis, and possible deformity of bone contour; and grade 4 = large osteophytes, marked joint space narrowing, severe sclerosis, and definite deformity of bone contour [23].

### 2.6 Outcome measures - MRI assessment of osteoarthritis

MR images at baseline and follow-up were assessed for osteoarthritic features using the University of California, San Francisco (UCSF)-modified Whole-Organ Magnetic Resonance Imaging Score (WORMS) [[24], [25], [26]]. Cartilage abnormalities were graded on a scale from 0 to 6 across six anatomical regions: patella, trochlea, medial femur, lateral femur, medial tibia, and lateral tibia. Meniscal pathology was evaluated using a grading scale from 0 to 4 in six subregions, defined by compartment (medial and lateral) and location (anterior horn, body, and posterior horn). Bone marrow edema-like lesions (BMELL) were graded on a semiquantitative scale ranging from 0 to 3 [25,26]. Effusion-synovitis was not included in the WORMS assessment performed in this study and was instead evaluated using MOAKS (see above). The two systems grade effusion-synovitis similarly, on the basis of the estimated maximal distention of the synovial cavity [13,24].

For statistical analyses, composite scores for overall WORMS, BMELL, cartilage, and meniscal damage were calculated by summing the corresponding scores across all evaluated regions, yielding a composite index of overall knee structural damage. Although these features are graded on different ordinal scales, this unweighted summation to derive a global WORMS score follows prior studies using data from the OAI [27].

### 2.7 Outcome measures - assessment of clinical symptoms

Knee symptoms at baseline and follow-up were assessed on a 5-point scale using the Western Ontario and McMaster Universities Osteoarthritis Index (WOMAC), a validated and widely used questionnaire for assessing symptoms related to knee osteoarthritis. [28,29]. Scores of the subscales of WOMAC pain, stiffness, and physical function were summarized as “total score” for further analysis, as previously reported in the literature [30].

### 2.8 Statistical analysis

Statistical analyses were conducted using R (version 4.5.0; R Foundation for Statistical Computing, Vienna, Austria). Primary outcomes included longitudinal changes from baseline to 48-month follow-up in KL grade (ΔKL), total WORMS score and subscores for meniscus, BMELL, and cartilage (ΔWORMS_Total_, ΔWORMS_Meniscus_, ΔWORMS_BMELL_, and ΔWORMS_Cartilage_), as well as changes in WOMAC total score (ΔWOMAC). The primary exposure was change in effusion-synovitis volume (ΔESV).

Spearman's rank correlation coefficient was used to evaluate the correlation between ESV change (ΔESV) and semiquantitative MOAKS-based effusion-synovitis change (ΔMOAKS). ΔESV observations exceeding 3 times the interquartile range were visually inspected by a fellowship-trained musculoskeletal radiologist (AAM). No segmentation errors were identified, and these observations were therefore retained in the analyses.

For subsequent analyses, ΔESV was transformed using a signed logarithmic transformation to reduce the influence of extreme values without excluding observations, because the distribution of ΔESV was heavy-tailed. To facilitate comparison of effect sizes across outcomes, ΔESV and outcomes were standardized (z-transformed) prior to model fitting, so that effect sizes are expressed in outcome standard deviation units.

All regression models were adjusted for age, sex, race, and baseline body mass index. Linearity of the association between ΔESV and each outcome was evaluated by comparing models with a linear exposure term to models using a natural cubic spline for the exposure. Nested models with and without a spline term were compared using partial F-tests. Because evidence of nonlinearity was consistently observed across outcomes (Supplementary Table S2), all primary analyses modeled exposures using natural cubic splines with three degrees of freedom (df = 3).

The overall association between ΔESV and each outcome was assessed using a global F-test comparing models with and without the spline term, from which p-values were obtained. Effect sizes were summarized as the difference in model-predicted outcome comparing the 90th and 10th percentiles (interdecile difference, IDD) of ΔESV on the model scale, with covariates held constant. Uncertainty in IDD estimates was quantified using a case-resampling bootstrap with 2000 replicates. Percentile-based 95% confidence intervals (CIs) were derived from the bootstrap distributions. Because outcomes were z-transformed prior to model fitting, effect sizes were interpreted using conventional benchmarks as described by Cohen [31]. Additionally, the 10th and 90th percentiles of ΔESV defining the interdecile range were reported in their original units (mL), and the corresponding IDDs were expressed in the original units of each outcome.

To compare ΔESV with semiquantitative MOAKS-based effusion-synovitis change (ΔMOAKS), spline-based models were additionally fitted with z-transformed ΔMOAKS as the primary exposure, using the same covariate adjustment and spline specification as in the analyses with ΔESV as primary exposure. Differences in association strength between ΔESV and ΔMOAKS were evaluated using a paired case-resampling bootstrap, in which the ΔESV and ΔMOAKS models were refit within each bootstrap sample and the difference in IDD (ΔIDD = ΔESV minus ΔMOAKS) was computed. Percentile-based 95% CIs and a two-sided bootstrap p-value were obtained for this contrast.

Effect modification by sex was evaluated on the basis of reported sex differences in osteoarthritis progression [32,33] by including an interaction between sex and the spline-based ΔESV term in covariate-adjusted regression models. Evidence of interaction was assessed using a partial F-test comparing models with and without the interaction term. When a statistically significant interaction was observed, sex-specific associations were summarized by estimating model-predicted differences in outcome comparing the IDD of ΔESV on the model scale for females and males separately. To assess the robustness of the findings to model specification, sensitivity analyses were performed. First, all models were refit with ΔESV entered as a linear term, with associations summarized as standardized β coefficients. Second, the primary analyses were repeated across a range of spline degrees of freedom (df = 4 and df = 5). For the linear models and for each spline model, the comparison of ΔESV against ΔMOAKS was repeated using the same paired case-resampling bootstrap. Statistical significance was evaluated using a two-sided α level of 0.05.

## 3 Results

### 3.1 Participants and distribution of ΔESV across ΔMOAKS categories

The baseline demographic characteristics of the participants are summarized in Table 1. Median baseline ESV was 4.02 mL (interquartile range [IQR] 4.42). The median ΔESV over four years was 0.50 mL (IQR 3.18). Among 1682 participants with available MOAKS scores at baseline and follow-up, 449 (26.7%) experienced an increase in ΔMOAKS, 156 (9.3%) experienced a decrease, and 1077 (64.0%) had no change.

**Table 1:** **Participant demographics.** Continuous variables are reported as mean (standard deviation), whereas categorical variables are reported as frequency (percentage).

Variable	
**Age, years**	60.5 (9.0)
**Body mass index, kg/m^2^**	28.2 (4.7)
**Kellgren-Lawrence grade**	
Grade 0	1077 (43.6%)
Grade 1	492 (19.9%)
Grade 2	676 (27.4%)
Grade 3	224 (9.1%)
**Sex**	
Female	1509 (61.1%)
Male	960 (38.9%)
**Race**	
Asian	18 (0.7%)
Black	359 (14.5%)
White	2081 (84.3%)
Other	9 (0.4%)
Missing	2 (0.1%)

**Fig. 2:**
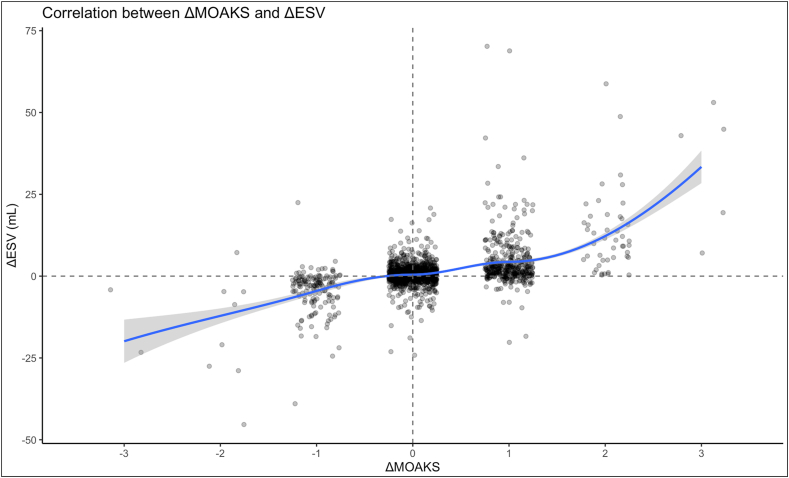
Association between change in quantitative effusion-synovitis volume (ΔESV) and change in MOAKS effusion-synovitis score (ΔMOAKS) from baseline to 48-month follow-up. Points represent individual participant observations, and the solid line denotes a locally estimated smoothing curve with 95% confidence bands.

**Fig. 3:**
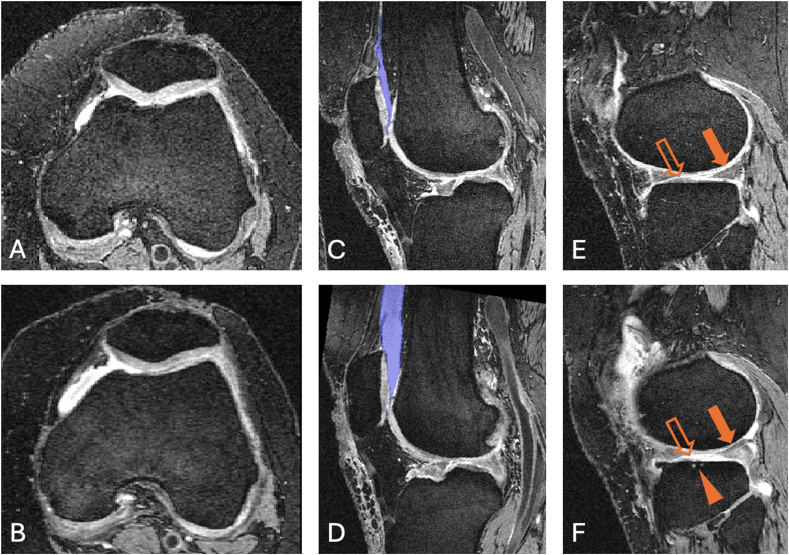
**Representative images illustrating the relationship between MRI Osteoarthritis Knee Score (MOAKS) gradings of effusion-synovitis change and quantitative assessment of effusion-synovitis volume (ESV) between baseline and follow-up.** MR images of the right knee of a 72-year-old female participant are shown. On axial dual-echo steady-state (DESS) images (A, B), effusion-synovitis was graded as “small” (MOAKS grade 1) at both baseline (A) and 48-month follow-up (B). In contrast, sagittal DESS images with overlaid masks of deep-learning-based effusion-synovitis segmentations demonstrate a marked increase from baseline (C) to follow-up (D), predominantly in the suprapatellar recess (quantified as a difference of 18.7 mL), which illustrates the limited granularity of the four-level ordinal scale of MOAKS. Sagittal DESS images of the lateral tibiofemoral joint at baseline (E) and follow-up (F) revealed progressive meniscal degeneration (arrow), cartilage loss at the tibial plateau (outline arrow), and new evidence of subchondral cystic changes of the tibial plateau (arrowhead). The total Whole-Organ Magnetic Resonance Imaging Score (WORMS) increased by 13 points over the follow-up period.

Spearman's rank correlation demonstrated a modest but statistically significant association between ΔESV and ΔMOAKS (ρ = 0.49; p < 0.001). Fig. 2 shows the association between ΔESV and ΔMOAKS, demonstrating a wide range of ΔESV values across ΔMOAKS categories. Representative MR images from a participant demonstrating an increase in ΔESV without a corresponding change in ΔMOAKS are shown in Fig. 3.

### 3.2 Association of ΔESV with OA outcomes

Spline-based regression models demonstrated a statistically significant overall association between ΔESV and all outcomes examined (ΔKL, ΔWORMS_Total_, ΔWORMS_Meniscus_, ΔWORMS_BMELL_, ΔWORMS_Cartilage_ and ΔWOMAC: p < 0.001; Table 2, Supplementary Figure S2). The magnitude of these associations, summarized using IDDs (90th vs 10th percentile of ΔESV), indicated moderate associations with higher ΔKL (IDD = 0.33, 95% CI 0.20 to 0.46), ΔWORMS_Total_ (IDD = 0.48, 95% CI 0.32 to 0.63), ΔWORMS_Meniscus_ (IDD = 0.38, 95% CI 0.24 to 0.52), ΔWORMS_Cartilage_ (IDD = 0.28, 95% CI 0.13 to 0.42) and ΔWOMAC (IDD = 0.36, 95% CI 0.24 to 0.47), whereas associations were weak for ΔWORMS_BMELL_ (IDD = 0.15, 95% CI 0.02 to 0.29) (Table 2). The IDDs are additionally presented in the original units of each outcome, alongside the corresponding 10th and 90th percentiles of ΔESV (in mL), in Supplementary Table S3.

**Table 2:** Associations between change in effusion-synovitis volume (ΔESV) and longitudinal changes in structural and clinical outcomes assessed using spline-based regression models. Effect sizes are presented as interdecile differences (IDDs), defined as the estimated difference in outcome between the 90th and 10th percentiles of ΔESV. Overall p-values correspond to tests of association between ΔESV and each outcome. In a comparative analysis, differences in association strength between ΔESV and change in MRI Osteoarthritis Knee Score (ΔMOAKS) were evaluated (ΔIDD = IDD_ΔESV_-IDD_ΔMOAKS_). Positive ΔIDD values indicate stronger associations for ΔESV. Estimates are reported with 95% confidence intervals (CIs).

Outcome	IDD	95% CI	p-value	ΔIDD (IDD_ΔESV_-IDD_ΔMOAKS)_	95% CI	p-value
ΔKL	0.33	0.20 to 0.46	**<0.001**	0.22	0.08 to 0.37	**0.002**
ΔWORMS total	0.48	0.32 to 0.63	**<0.001**	0.21	0.06 to 0.36	**0.008**
ΔWORMS meniscus	0.38	0.24 to 0.52	**<0.001**	0.20	0.03 to 0.33	**0.026**
ΔWORMS cartilage	0.28	0.13 to 0.42	**<0.001**	0.09	−0.06 to 0.25	0.25
ΔWORMS BMELL	0.15	0.02 to 0.29	**<0.001**	0.06	−0.09 to 0.24	0.51
ΔWOMAC	0.36	0.24 to 0.47	**<0.001**	0.18	−0.16 to 0.33	0.19

Bold values indicate statistically significant differences (p < 0.05).

When comparing ΔESV with ΔMOAKS, ΔESV demonstrated statistically significant stronger associations for ΔKL (ΔIDD = 0.22, 95% CI 0.08 to 0.37; p = 0.002), ΔWORMS_Total_ (ΔIDD = 0.21, 95% CI 0.06 to 0.36; p = 0.008), and ΔWORMS_Meniscus_ (ΔIDD = 0.20, 95% CI 0.03 to 0.33; p = 0.026). Differences in association strength were higher for ΔESV but not statistically significant for ΔWORMS_BMELL_ (ΔIDD = 0.06, 95% CI -0.09 to 0.24; p = 0.51), ΔWORMS_Cartilage_ (ΔIDD = 0.09, 95% CI -0.06 to 0.25; p = 0.25), or ΔWOMAC (ΔIDD = 0.18, 95% CI -0.16 to 0.33; p = 0.19). In sensitivity analyses, ΔESV remained significantly associated with all outcomes when using a linear model (standardized β 0.06–0.20; all p ≤ 0.031; Supplementary Table S4), and in spline-based models at higher degrees of freedom (Supplementary Table S5). However, the comparative analysis was sensitive to model specification: the stronger associations of ΔESV relative to ΔMOAKS were no longer statistically significant in linear models or spline-based models with higher spline degrees of freedom (Supplementary Table S4+S5).

**Fig. 4:**
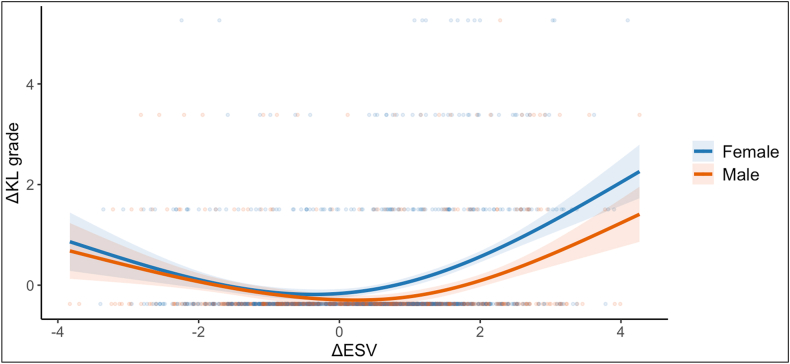
**Association between change in effusion-synovitis volume (ΔESV) and change in Kellgren-Lawrence grade (ΔKL), stratified by sex.** A significant interaction between sex and the spline-based ΔESV term was observed (global interaction p = 0.005). The association was stronger in females, with a larger interdecile difference compared with males; the between-sex difference in interdecile differences (ΔIDD), estimated using bootstrap resampling, was 0.44 (95% CI 0.18–0.69; p < 0.001). Both ΔESV and ΔKL are shown on standardized scales. Points represent observed standardized ΔKL values, solid lines indicate model-predicted means, and shaded bands denote 95% confidence intervals.

Effect modification by sex was observed for ΔKL (global interaction p = 0.005; Fig. 4). Among women, the IDD in ΔKL associated with ΔESV was larger (IDD = 0.64, 95% CI 0.48 to 0.81) than among men (IDD = 0.21, 95% CI 0.01 to 0.40), indicating a stronger association in women (ΔIDD = 0.44, 95% CI 0.18 to 0.69; p < 0.001).

## 4 Discussion

This study investigated whether the change of deep learning-based effusion-synovitis volume (ΔESV) on MR images is associated with radiographic, MRI-based, and symptomatic OA progression using data from the OAI between baseline and 48 months. Moreover, we compared the strength of the association between ΔESV and the change in semiquantitative effusion-synovitis, as measured by the MRI Osteoarthritis Knee Score (ΔMOAKS). We found that ΔESV was significantly associated with all measures of OA progression. Stronger associations with radiographic and MRI-based progression (total score of the Whole-Organ Magnetic Resonance Imaging Score [WORMS]) were observed for ΔESV compared to ΔMOAKS. A significant sex-based effect modification was observed for radiographic OA progression, with stronger associations in women.

The observed significant associations between ESV change used as an imaging surrogate of synovitis and OA outcomes align with current evidence indicating that synovial inflammation is associated with OA progression [34]. Synovial inflammation is thought to be mediated by various factors, such as activation of the innate immune system, mechanical stress, metabolic factors, and neurovascular signaling, resulting in hyperplasia, fibrosis, and angiogenesis of the synovial tissue [[35], [36], [37]]. This transforms the secreted synovial fluid into a pro-inflammatory environment enriched with metabolites, chemokines, growth factors, and cytokines that amplify inflammatory signaling and promote tissue damage [37]. Because synovial fluid mediates bidirectional communication between joint tissues, these processes can establish a self-perpetuating inflammatory loop that accelerates cartilage degradation and pathological tissue remodeling [38,39].

Our results are consistent with previous studies demonstrating an association between effusion-synovitis and structural OA progression, as assessed using semiquantitative scoring on non-contrast-enhanced MRI [[6], [7], [8], [9]]. Non–contrast-enhanced MRI is the preferred non-invasive method that is widely used in OA research for assessment of effusion-synovitis, primarily due to both the safety considerations and the economic burden associated with intravenous contrast agents [24,40,41]. Although semiquantitative scoring systems such as the MOAKS are well validated and widely used for the assessment of effusion-synovitis, they are not without limitations. These methods rely on interpretation by expert readers, are time-consuming, and have moderate sensitivity for detecting change, especially when readers are blinded to the different imaging acquisition time points [[14], [15], [16]]. Consequently, the stronger associations observed for ΔESV than for ΔMOAKS in imaging-based OA progression suggest that ΔESV may be a more attractive alternative to semiquantitative scoring.

Another advantage is that ESV can be quantified using fully automated deep learning algorithms, which have demonstrated promising performance in preliminary research [42,43]. The deep learning algorithm applied here has been described previously for thigh muscle segmentation [21] and was evaluated for the segmentation of effusion-synovitis in internal validation experiments [19], achieving acceptable agreement with manual annotations, with a Dice coefficient of 0.79. The comparatively weak association observed between ΔESV and ΔWORMS_BMELL_ may reflect the dynamic and potentially reversible nature of BMELL, which can fluctuate over time, in contrast to other MRI-based structural WORMS features [44].

While the association between synovitis and knee pain is well established in various cross-sectional studies [10,11], our finding that ΔESV is associated with symptom progression further corroborates longitudinal evidence demonstrating worsening symptoms with increasing extent of effusion-synovitis [45,46]. The underlying mechanisms by which synovitis contributes to knee OA pain proposed by the current literature include pro-inflammatory mediator release, innate immune activation, neurovascular invasion, and central nociceptive sensitization [34,35].

Notably, a sex interaction was observed in the association between ESV and radiographic OA progression, with a significantly stronger association in females. Sex-dependent differences in structural OA progression have been reported previously [33], and prior work by Roemer et al. demonstrated that the presence of effusion-synovitis was associated with a greater risk of incident radiographic OA in overweight women [32]. These observations highlight the need for future longitudinal studies to incorporate sex-specific analyses when evaluating synovitis as a biomarker of disease trajectories.

It should be considered that the volumetric changes in effusion-synovitis observed in the study were small (median ΔESV 0.50 mL; interdecile range approximately −3 to +7 mL). A joint effusion of this size would most likely be below the threshold for reliable detection on physical examination, which identifies only moderate-to-large joint effusion [12]. Ultrasonography, although more sensitive than clinical examination for detecting joint effusion, remains operator-dependent and has limited value for volumetric effusion quantification [47]. Because synovitis is considered a modifiable risk factor for OA progression, and given the limitations of other modalities and physical examination described above, the present findings highlight ΔESV as a promising imaging biomarker for therapeutic targeting. Future longitudinal studies could examine whether ΔESV is responsive to anti-inflammatory oral medication or intra-articular steroid therapy.

This study is not without limitations. First, there was a lack of a reference standard for ESV quantification based on non-contrast-enhanced MRI. Because synovial tissue and joint fluid demonstrate similar signal intensity on the DESS sequence, segmentation may have inadvertently included synovial tissue, potentially inflating estimates of true joint effusion. Consistent with prior methodological recommendations for non-contrast-enhanced MRI in large cohort studies, we therefore described this measure as “effusion-synovitis” [13]. Second, while the associations between ΔESV and all outcomes were robust across both linear and spline models and across a range of spline degrees of freedom, the comparative analysis indicating stronger associations for ΔESV than for ΔMOAKS was sensitive to model specification. The superiority of ΔESV over ΔMOAKS should therefore be interpreted with caution. Additionally, multiple outcomes and exposure comparisons were examined without formal correction for multiple testing. However, the consistency and robustness of the primary ΔESV associations across models and sensitivity analyses support the validity of the observed associations. Third, we acknowledge that a continuous exposure such as ΔESV can show stronger statistical associations than an ordinal measure such as ΔMOAKS simply through greater scalability, independent of clinical relevance, which remains to be established. Finally, we evaluated ΔESV between the available examinations from baseline and 48-month follow-up, whereas short-term or transient fluctuations in ESV occurring within this interval may have been missed.

In summary, this study demonstrated that ΔESV from baseline to 48-month follow-up is associated with concurrent measures of both imaging-based and knee symptom progression of knee OA. The stronger associations of ΔESV compared to ΔMOAKS with imaging-based OA outcomes underscore the potential of ΔESV as a quantitative OA imaging biomarker, although further studies are needed to evaluate its predictive and clinical utility.

## Author contributions

The authors have made substantial contributions to the following sections:

•Conception and design (AAM TML GBJ)
•Analysis and interpretation of the data (AAM FL NEL CEM MCN TML GBJ)
•Collection and assembly of data (AAM FL EP TML GBJ)
•Drafting of the article (AAM)
•Statistical expertise (GBJ CEM)
•Critical revision of the article for important intellectual content (AAM FL EP STU JAL ASG NEL MCN CEM TML GBJ)
•Final approval of the article (AAM FL EP STU JAL ASG NEL MCN CEM TML GBJ)

## Role of the funding source

This study was funded by 10.13039/100000002NIH R01-AR064771, 10.13039/100000002NIH R01-AR078917, and NIH R01-AG070647. Additionally, this work was supported in part by CCMBM P30AR075055. The 10.13039/100019120OAI is a public-private partnership comprised of five contracts (N01-AR-2-2258; N01-AR-2-2259; N01-AR-2-2260; N01-AR-2-2261; N01-AR-2-2262) funded by the 10.13039/100000002National Institutes of Health, a branch of the Department of Health and Human Services, and conducted by the 10.13039/100019120OAI Study Investigators. Private funding partners include 10.13039/100004334Merck Research Laboratories; 10.13039/100008272Novartis Pharmaceuticals Corporation, GlaxoSmithKline; and 10.13039/100004319Pfizer, Inc. Private sector funding for the 10.13039/100019120OAI is managed by the 10.13039/100000009Foundation for the National Institutes of Health.

## Competing interests

TML serves as an Associate Editor for Osteoarthritis and Cartilage Open. The remaining authors declare no competing financial or personal interests related to this work.
