# Supplementary material for: Deep learning-based effusion-synovitis volume measured on MRI is associated with osteoarthritis progression: A longitudinal analysis of data from the osteoarthritis initiative
Source: Osteoarthr Cartil Open. 2026 Jun 27;8(3):100847. doi: 10.1016/j.ocarto.2026.100847 (PMC13355676; doi:10.1016/j.ocarto.2026.100847)
Supplement: Multimedia component 1 [file mmc1.docx]

**Supplementary Material**

**Supplementary Table S1. Deep Learning Model Pipeline for Knee Effusion-Synovitis Segmentation.**

| **Category** | **Subcategory** | **Description** |
| --- | --- | --- |
| Dataset | Source | Osteoarthritis Initiative (https://nda.nih.gov/oai) |
|  | Total annotated knees | 101 right knees from baseline data |
|  | MRI sequence | Sagittal 3D dual-echo steady-state (DESS) with water excitation |
|  | Annotators | Radiology resident and trained student operator under the supervision of a musculoskeletal radiologist with >20 years of experience |
|  | Annotation software | MD.ai (MD.ai, Inc.) |
|  | Inter-reader reliability subset | 16 knees |
| Data Split | Training set | 80 knees |
|  | Validation set | 10 knees |
|  | Test set | 11 knees |
| Detection Model (YOLOv11) | Model type | YOLOv11 (You Only Look Once, version 11, ultralytics.com) deep learning object detection model |
|  | Training labels | Bounding boxes derived from manual segmentations |
|  | Performance | Mean Intersection-over-Union = 0.85 (95% CI: 0.84-0.87) |
|  | Detection accuracy | Mean Average Precision = 0.86 |
| Segmentation Model (SAM) | Model type | Segment Anything Model (SAM, version 1, ai.meta.com), foundation model for prompt-based segmentation |
|  | Input prompt | Bounding boxes from YOLOv11 |
|  | Performance | Dice coefficient = 0.79 (95% CI: 0.68-0.87) |
| Final Pipeline |  | YOLOv11 detection → SAM segmentation → subtraction of available cartilage, meniscus, and bone masks from previous studies |
|  | Final performance | Dice coefficient = 0.79 (95% CI: 0.71-0.86) |
| Benchmark Models | 2D nnU-Net | Dice coefficient = 0.68 (95% CI: 0.61-0.75) |
|  | 3D nnU-Net | Dice coefficient = 0.74 (95% CI: 0.66-0.81) |
| Inter-reader Agreement | Dice coefficient | 0.67 (95% CI: 0.60-0.72) |

CI, Confidence interval; DESS, Dual-echo steady-state; YOLO, You Only Look Once; SAM, Segment Anything Model

**Supplementary Table S2.** **Tests of Nonlinearity for ΔESV**. Partial F-tests comparing a linear ΔESV term with a natural cubic spline (df = 3). For all outcomes, the natural cubic spline model provided a significantly better fit than the corresponding linear model (all p < 0.001). Outcomes were assessed as the change (Δ) over 48-month follow-up period.

| **Outcome** | **F statistic** | **p-value** |
| --- | --- | --- |
| Δ KL grade | 57.70 | <0.001 |
| Δ WORMS Total | 50.39 | <0.001 |
| Δ WORMS Meniscus | 14.21 | <0.001 |
| Δ WORMS Cartilage | 47.27 | <0.001 |
| Δ WORMS BMELL | 22.48 | <0.001 |
| Δ WOMAC | 8.68 | <0.001 |

BMELL, bone marrow edema-like lesions; ESV, effusion-synovitis volume; KL, Kellgren-Lawrence; WOMAC, Western Ontario and McMaster Universities Osteoarthritis Index; WORMS, Whole-Organ Magnetic Resonance Imaging Score.

**Supplementary Table S3.** Effect estimates in original outcome units. For each outcome, the 10th and 90th percentiles of ΔESV (in mL) define the interdecile range over which the effect is estimated. Effect sizes from the primary spline models were presented as interdecile differences (IDDs), defined as the predicted difference in outcome between the 90th and 10th percentiles of ΔESV, expressed as the change of the original unit of each outcome.

| **Outcome** | **ΔESV 10th percentile (mL)** | **ΔESV 90th percentile (mL)** | **IDD (95% CI)** |
| --- | --- | --- | --- |
| ΔKL grade | -3.02 | 6.67 | 0.18 (0.11 to 0.25) |
| ΔWORMS Total | -3.18 | 7.00 | 2.55 (1.70 to 3.34) |
| ΔWORMS Meniscus | -3.17 | 6.63 | 0.68 (0.43 to 0.93) |
| ΔWORMS Cartilage | -3.18 | 6.62 | 0.78 (0.36 to 1.16) |
| ΔWORMS BMELL | -3.18 | 6.64 | 0.24 (0.03 to 0.47) |
| ΔWOMAC Total | -3.12 | 6.68 | 4.24 (2.83 to 5.53) |

BMELL, bone marrow edema-like lesions; CI, confidence interval; ESV, effusion-synovitis volume; IDD, interdecile difference; KL, Kellgren-Lawrence; WOMAC, Western Ontario and McMaster Universities Osteoarthritis Index; WORMS, Whole-Organ Magnetic Resonance Imaging Score.

**Supplementary Table S4. Sensitivity analysis using multivariable linear regression.** Change in effusion-synovitis volume (ΔESV) was entered as a linear term predicting each outcome, adjusted for age, sex, race, and body mass index. Outcomes were assessed as the change (Δ) over 48-month follow-up period. Standardized β coefficients with 95% Confidence Intervals (CIs) represent the standard deviation (SD) change in outcome per 1-SD increase in ΔESV. In a comparative analysis, differences in association strength between ΔESV and change in MRI Osteoarthritis Knee Score (ΔMOAKS) were evaluated. Positive Δβ values indicate stronger associations for ΔESV.

| **Outcome** | **β (95% CI)** | **p-value** | **Δβ (β_ESV_ - β_MOAKS_) (95% CI)** | **p-value** |
| --- | --- | --- | --- | --- |
| Δ KL grade | 0.17 (0.12 to 0.21) | <0.001 | 0.04 (-0.01 to 0.09) | 0.143 |
| Δ WORMS Total | 0.20 (0.15 to 0.25) | <0.001 | 0.02 (-0.04 to 0.07) | 0.560 |
| Δ WORMS Meniscus | 0.17 (0.12 to 0.22) | <0.001 | 0.03 (-0.02 to 0.08) | 0.189 |
| Δ WORMS Cartilage | 0.11 (0.06 to 0.16) | <0.001 | 0.00 (-0.05 to 0.05) | 0.925 |
| Δ WORMS BMELL | 0.06 (0.01 to 0.11) | 0.031 | 0.00 (-0.05 to 0.06) | 0.901 |
| Δ WOMAC Total | 0.20 (0.15 to 0.25) | <0.001 | 0.00 (-0.06 to 0.05) | 0.899 |

BMELL, bone marrow edema-like lesions; CI, confidence interval; ESV, effusion-synovitis volume; KL, Kellgren-Lawrence; MOAKS, MRI Osteoarthritis Knee Score; SD, standard deviation; WOMAC, Western Ontario and McMaster Universities Osteoarthritis Index; WORMS, Whole-Organ Magnetic Resonance Imaging Score.

**Supplementary Table S5.** **Sensitivity analysis evaluating the influence of the spline degrees of freedom (df) on the associations between ΔESV and outcomes and on the comparative analysis (ΔESV / ΔMOAKS).** Outcomes were assessed as the change (Δ) over 48-month follow-up period. As in the primary analysis (Table 2), effect sizes are presented as interdecile differences (IDDs), defined as the estimated difference in outcome between the 90th and 10th percentiles of ΔESV. The comparative analysis evaluated the difference in association strength between ΔESV and ΔMOAKS (ΔIDD = IDDΔ_ESV_ - IDDΔ_MOAKS_). Positive ΔIDD values indicate stronger associations for ΔESV. Models were adjusted for age, sex, race, and body mass index.

R1.4

| **Outcome** | **IDD (95% CI), df = 4** | **p-value** | **ΔIDD (95% CI), df = 4** | **p-value** | **IDD (95% CI), df = 5** | **p-value** | **ΔIDD (95% CI), df = 5** | **p-value** |
| --- | --- | --- | --- | --- | --- | --- | --- | --- |
| Δ KL | 0.45 (0.30 to 0.61) | <0.001 | 0.12 (-0.06 to 0.28) | 0.177 | 0.45 (0.28 to 0.61) | <0.001 | 0.10 (-0.07 to 0.28) | 0.274 |
| Δ WORMS Total | 0.50 (0.34 to 0.67) | <0.001 | 0.15 (-0.01 to 0.31) | 0.067 | 0.51 (0.33 to 0.69) | <0.001 | 0.14 (-0.05 to 0.31) | 0.138 |
| Δ WORMS Meniscus | 0.41 (0.27 to 0.56) | <0.001 | 0.15 (-0.05 to 0.30) | 0.131 | 0.40 (0.25 to 0.55) | <0.001 | 0.14 (-0.06 to 0.30) | 0.143 |
| Δ WORMS Cartilage | 0.26 (0.11 to 0.42) | <0.001 | 0.04 (-0.12 to 0.21) | 0.619 | 0.27 (0.10 to 0.43) | <0.001 | 0.03 (-0.15 to 0.23) | 0.760 |
| Δ WORMS BMELL | 0.14 (0.02 to 0.30) | <0.001 | 0.00 (-0.17 to 0.20) | 0.978 | 0.14 (0.02 to 0.30) | <0.001 | -0.01 (-0.20 to 0.23) | 0.894 |
| Δ WOMAC Total | 0.41 (0.27 to 0.55) | <0.001 | 0.14 (-0.19 to 0.31) | 0.225 | 0.40 (0.27 to 0.54) | <0.001 | 0.13 (-0.21 to 0.30) | 0.251 |

BMELL, bone marrow edema-like lesions; CI, confidence interval; ESV, effusion-synovitis volume; IDD, interdecile difference; KL, Kellgren-Lawrence; WOMAC, Western Ontario and McMaster Universities Osteoarthritis Index; WORMS, Whole-Organ Magnetic Resonance Imaging Score.

**Supplementary Figure S1. Representative examples of manual and automated effusion-synovitis segmentation on sagittal dual-echo steady-state (DESS) MRI of the right knee.**

In the upper row (A, B), a representative image slice of a segmentation case with good agreement between manual reference segmentations (A) and automated deep-learning based segmentations (B) highlighted red is demonstrated (Dice coefficient: 0.89). In the lower row (C, D), a representative slice of a case with erroneous segmentation is presented (Dice coefficient: 0.72). (C) shows the manual reference segmentation, while (D) shows the corresponding automated segmentation, demonstrating under-segmentation with missing coverage of the inferior effusion-synovitis from the manual annotation.


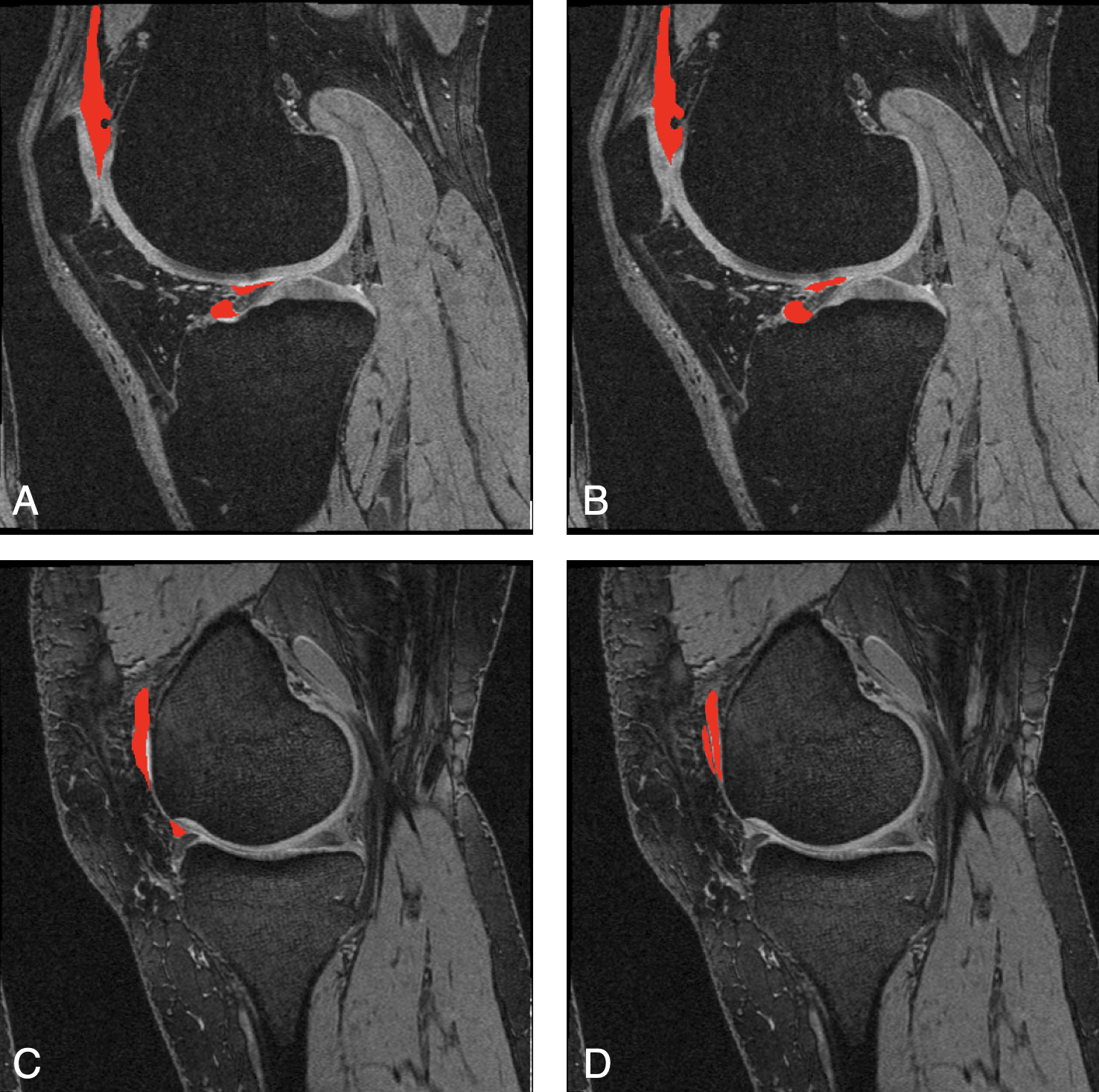


**Supplementary Figure S2.** **Associations between change in effusion-synovitis volume (ΔESV) and each outcome using natural cubic spline regression.** In each panel, the solid line denotes the change in standardized (z-transformed) outcome (in standard deviation [SD] units) across the range of ΔESV, and the shaded area represents the corresponding 95% confidence band.


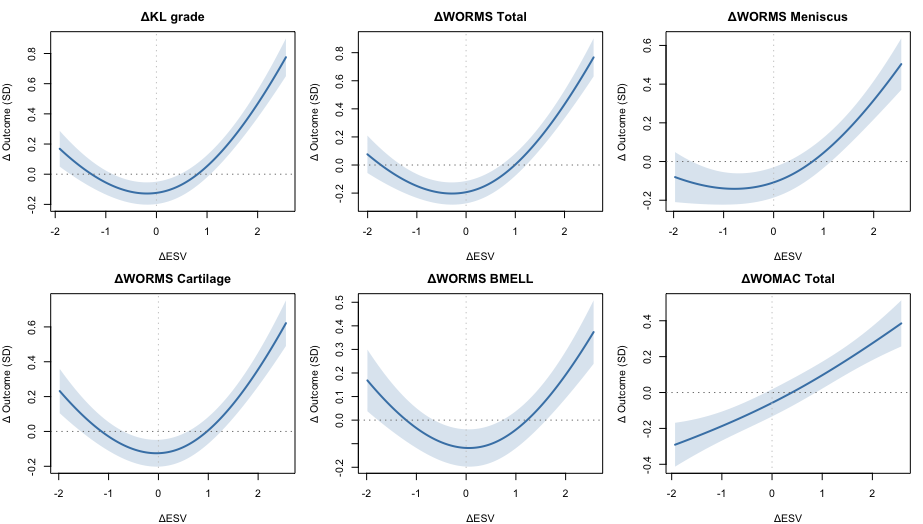


BMELL, bone marrow edema-like lesions; ESV, effusion-synovitis volume; KL, Kellgren-Lawrence; SD, standard deviation; WOMAC, Western Ontario and McMaster Universities Osteoarthritis Index; WORMS, Whole-Organ Magnetic Resonance Imaging Score.
